# An exploration of the 3D chemical space has highlighted a specific shape profile for the compounds intended to inhibit protein-protein interactions

**DOI:** 10.1186/1471-2105-16-S3-A5

**Published:** 2015-02-13

**Authors:** Mélaine A Kuenemann, Laura ML Bourbon, Céline M Labbé, Bruno O Villoutreix, Olivier Sperandio

**Affiliations:** 1 Université Paris Diderot, Sorbonne Paris Cité, UMRS 973 Inserm, Paris 75013, France; 2 Inserm, U973, Paris 75013, France; 3 CDithem, Faculté de Pharmacie, 1 rue du Prof Laguesse, 59000 Lille, France

## Background

The vital role of Protein-Protein Interactions (PPI) for Life makes them the subject of a growing number of drug discovery projects. Yet, the specific properties of PPI (often described as flat, large and hydrophobic) require a dramatic paradigm shift in our way to design the small compounds meant to modulate them with therapeutic perspectives. To this end, successful inhibitors of PPI targets (iPPI) may be used to discover what singular properties make this type of inhibitors capable of binding to such intricate surfaces. Among the properties from which lessons could be learnt, the 3D characteristics of iPPI have been pinpointed as essential. Understanding the putative shape profile of iPPI could help the design of a new generation of inhibitors.

## Results

**Figure 1:**
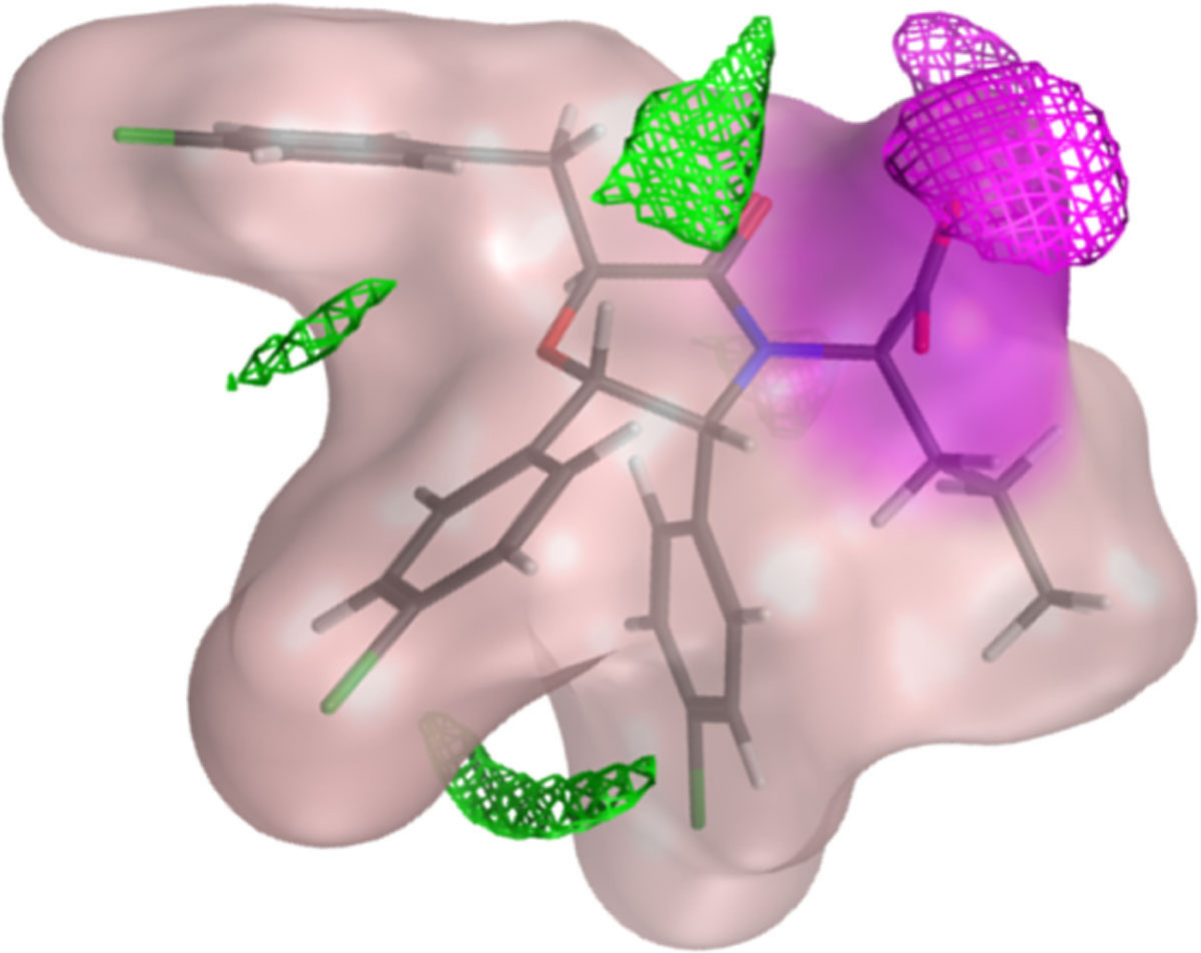
**Bioactive conformation of compound 1MQ as cocrystallized with Mdm2 (pdb code **4JVE**)**. The compound is represented as transparent molecular surface and molecular sticks. The value of highlighted descriptors are : EDmin3 = -3.18 kcal/mol (represented by the green molecular field calculated using Moe 2012.10 at the levels of energy equal to -2.4 kcal/mol using a dry probe), IW4 = 4.13 (represented by the pink molecular field calculated using Moe 2012.10 at the levels of energy equal to -5.5 kcal/mol using a water probe), glob = 0.20 (represented by the molecular surface), and CW2 = 1.90 (represented by the proportion of pink surface over the full molecular surface).

In an attempt to identify 3D characteristics, we have collected the bioactive conformations of 84 orthosteric iPPI and compared them to those of 1282 inhibitors of conventional targets (e.g enzymes) collectively from different databases (2P2I[1], PDBbind[2], PDB). Because the known heavier and more hydrophobic character of iPPI could conceal other characteristics, we have imposed that none of the identified descriptors could correlate with the hydrophobicity or the size of the compound. Four 3D characteristics were highlighted (Figure 1). They describe either the shape of the compounds (globularity) or the 3D distributions of the hydrophobic and hydrophilic interacting regions of the compounds (IW4, EDmin3, CW2: VolSurf descriptors [3]). More specifically the most essential property revealed in the analysis (EDmin3) illustrates how iPPI manage to bind to the hydrophobic patches often present at the core of PPI targets. The newly identified properties were further confirmed as characteristic to iPPI using the data of much larger datasets including our iPPI-DB[4], eDrugs3D[5] and a representative subset of the bindingDB[6].

## Conclusions

Identifying low-molecular-weight iPPI is known to be a difficult task. This has usually been translated into designing compounds with higher size, aromaticity, and hydrophobicity. Yet, lessons are being learnt from iPPI bioactive conformations in an attempt to circumvent this trend. During this analysis, we demonstrated that the capacity to bind a protein-protein interface partially rely on the combination of several structural and electrostatic features including the globularity and the distribution of hydrophilic regions but most importantly of hydrophobic interacting regions. More distinctively, iPPI seem to be characterized by a significantly higher efficiency to bind the hydrophobic patches often present at PPI interfaces. The absence of correlation of this type of property with the hydrophobicity and the size of the compounds could open new ways to design iPPI with improved ligand and lipophilic efficiencies and may allow the scientific community to anticipate an era of more drug-like iPPI.
